# Topographic characteristics after Descemet’s membrane endothelial keratoplasty and Descemet’s stripping automated endothelial keratoplasty

**DOI:** 10.1371/journal.pone.0188832

**Published:** 2017-11-30

**Authors:** Takahiko Hayashi, Takefumi Yamaguchi, Kentaro Yuda, Naoko Kato, Yoshiyuki Satake, Jun Shimazaki

**Affiliations:** 1 Department of Ophthalmology, Yokohama Minami Kyosai Hospital, Kanagawa, Japan; 2 Department of Ophthalmology, Yokohama City University School of Medicine, Kanagawa, Japan; 3 Department of Ophthalmology, Jichi Medical University, Tochigi, Japan; 4 Department of Ophthalmology, Saitama Medical University, Saitama, Japan; 5 Department of Ophthalmology, Tokyo Dental College, Ichikawa General Hospital, Chiba, Japan; Save Sight Institute, AUSTRALIA

## Abstract

**Purpose:**

To investigate the topographic characteristics of the posterior corneal surface after Descemet’s endothelial membrane keratoplasty (DMEK) and Descemet’s stripping automated endothelial keratoplasty (DSAEK) and their effects on postoperative visual acuity.

**Methods:**

Nineteen eyes of 19 patients after DMEK, 23 eyes of 23 patients after DSAEK, and 18 eyes of 18 control subjects were retrospectively analyzed. Best spectacle-corrected visual acuity (BSCVA), aberration factors (higher-order aberrations [HOAs], spherical aberrations [SAs], and coma aberrations [Comas] at 6.0 mm) were evaluated preoperatively and at 1, 3, and 6 months postoperatively. The posterior refractive pattern of the topography map was classified into 5 grades (0–5) (posterior color grade) using anterior segment optical coherence tomography. Correlations between BSCVA and some factors (abbreviation factors, posterior color grade) were analyzed.

**Results:**

BSCVA was significantly better after DMEK than after DSAEK (*P* < 0.001). Posterior HOAs, SAs, and Comas after each type of endothelial keratoplasty were significantly greater compared to control (*P* < 0.01). Posterior HOAs, total/anterior/posterior SAs, and posterior color grade were significantly lower in the DMEK group than in the DSAEK group at 3 months (*P* < 0.024 [posterior HOAs], *P* = 0.047 [total SA], *P* < 0.001 [anterior SAs], *P* = 0.021 [posterior SAs], and *P* < 0.001 [posterior color grade]) and 6 months postoperatively (*P* = 0.034 [posterior HOAs], *P* < 0.001 [total SAs], *P* < 0.001 [anterior SAs], *P* = 0.013 [posterior SAs], and *P* = 0.004 [posterior color grade]). BSCVA was significantly correlated with HOAs, SAs, and posterior color grade (*P* < 0.001 for all except anterior HOAs [*P* = 0.004]).

**Conclusions:**

High posterior color grades were associated with larger aberration factors and had a negative effect on visual function after endothelial keratoplasty. Rapid improvement of visual function after DMEK may be attributed to less change at the posterior surface.

## Introduction

In recent times, there have been rapid advances in the surgical techniques used for endothelial keratoplasty (EK), and Descemet’s stripping automated endothelial keratoplasty (DSAEK) and Descemet’s membrane endothelial keratoplasty (DMEK) are now performed worldwide.[1–3] In comparison with penetrating keratoplasty, DSAEK and DMEK have advantages in terms of causing less astigmatism, having a lower risk of rejection, and allowing rapid visual recovery. However, recent studies suggest that best spectacle-corrected visual acuity (BSCVA) is generally better after DMEK than after DSAEK, probably because of less interface haze, folds in the donor disc, disc decentration, and problems related to thickness.[4–9]

As with DSAEK, we sometimes encounter patients with poor visual acuity of around 20/40–20/25 after DMEK despite excellent corneal clarity. Few studies have investigated the mechanisms determining quality of vision after DMEK.[4, 5] The reasons for poor visual acuity after EK (DMEK or DSAEK) are reported to be forward scattering,[5] corneal higher-order aberrations (HOAs), and interface haze.[6] Paradoxically, EK replaces the corneal endothelium, which presumably alters the curvature of the posterior corneal surface. However, HOAs of the anterior corneal surface have a greater influence on HOAs of the total cornea and visual acuity after EK.[7,8] The influence of HOAs on visual acuity is still poorly understood in eyes after DMEK.

Wavefront analyses to quantify lower-order aberrations and HOAs have explained the decreased visual acuity and contrast sensitivity in normal eyes [10] and in eyes with a number of disorders.[11–14] Our group has recently demonstrated elevated HOAs of the posterior cornea caused by an irregular surface in various diseases of the corneal surface[15–18] and found a significant correlation between corneal HOAs and visual acuity in patients.

**Fig 1:**
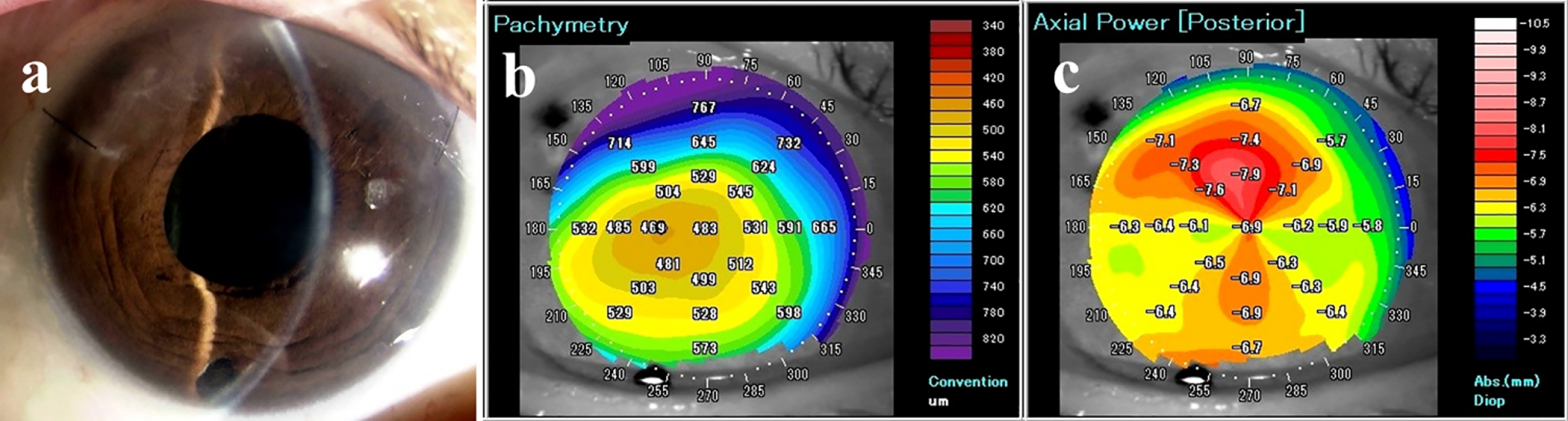
A patient with poor visual acuity and high posterior color grade 3 months after DMEK. (A) Slit-lamp photograph shows high transparency 3 months after DMEK. (B) Pachymetry after DMEK shows that the central corneal thickness is about 480 μm, which is considerably thinner than a healthy cornea. (C) Posterior map using AS-OCT after DMEK. Three months postoperatively, AS-OCT shows rapid improvement in corneal edema after DMEK. However, despite the clear cornea after DMEK, the visual acuity is 20/40. In this case, the posterior color grade is relatively high (grade 2). The figure demonstrates the characteristically irregular topography and increased irregular astigmatism at the posterior corneal surface in eyes with relatively poor visual acuity after DMEK. Abbreviations: AS-OCT, anterior segment optical coherence tomography; DMEK, Descemet’s endothelial membrane keratoplasty.

We have also noticed a characteristic irregular topography and increased irregular astigmatism at the posterior corneal surface in eyes with relatively poor visual acuity after DMEK (Fig 1). We hypothesized that corneal HOAs of the posterior surface might contribute to poor visual acuity after EK. The aims of this study were to measure corneal HOAs after DSAEK and DMEK, to compare them with those in normal eyes, and to evaluate the relationship between corneal HOAs and visual acuity after EK. Further, to the best of our knowledge, this is the first report that classified the topographic patterns of the posterior corneal surface into 6 grades that have a correlation with postoperative visual acuity and are easy to categorize.

## Patients and methods

This retrospective study followed the ethical standards of the Declaration of Helsinki and was approved by the institutional review board at Yokohama Minami Kyosai Hospital (approval number_28_3_1). Our Institutional Review Board waived the requirement for informed consent for this retrospective study. Patient data were anonymized before access and/or analysis.

### Patients

The study included 19 eyes undergoing DMEK (4 men, 15 women, mean age 72.2 ± 8.2 years) and 23 eyes undergoing DSAEK (5 men, 18 women, mean age 73.7 ± 5.9 years). The disorders in each group included Fuchs’ endothelial corneal dystrophy (FECD, n = 4 in the DMEK group, n = 6 in the DSAEK group), bullous keratopathy (BK) caused by argon laser iridotomy (ALI, n = 8 in the DMEK group, n = 10 in the DSAEK group), and pseudophakic bullous keratopathy (PBK, n = 7 in each group). The control group comprised 18 age-matched patients with normal phakic eyes (6 men, 12 women, mean age 74.8 ± 6.7 years) with no history of ocular disease or surgery. We excluded patients with pre-existing conditions limiting visual acuity, including macular degeneration, diabetic cystoid macular edema, retinal vein occlusion with cystoid macular edema, amblyopia, and end-stage glaucomatous optic atrophy. Table 1 shows the preoperative demographic and clinical characteristics of the DSAEK, DMEK, and control groups. Although both groups of patients were treated around the same time, the type of surgery was selected based on some factors such as patient social background. For example, we selected DSAEK for those patients who dislike the rebubbling procedure. Because Asian eyes present ALI with severe corneal edema, the central corneal thickness of the DMEK group was over 700 μm. Anterior segment optical coherence tomography (AS-OCT) (Casia SS-1000) has been used. AS-OCT the images were routinely taken in all patients with corneal dystrophies.

**Table 1:** Patient demographic and clinical characteristics before surgery.

	Control	DMEK	DSAEK	P*	P^†^ ^DMEK vs CT^	P^†^ ^DSAEK vs CT^	P^†^ ^DMEK vs DSAEK^
Eyes (n)	18	19	23				
Sex (male/female)	6/12	4/15	5/18	0.644*			
Age	74.8	72.2	73.6	0.462^†^			
Eye (R/L)	14/4	13/6	14/9	0.513*			
BSCVA (logMAR)	-0.03	1.01 ± 0.55^††^	1.28 ± 0.43^††^	< 0.001	< 0.001	< 0.001	NS
CCT	527± 27	714 ± 99^††^	759 ± 130^††^	<0.001	< 0.001	< 0.001	NS

BSCVA, best spectacle-corrected visual acuity; CCT, central corneal thickness; DMEK, Descemet’s endothelial membrane keratoplasty; DSAEK, Descemet’s stripping automated endothelial keratoplasty; ECD, endothelial cell density; logMAR, logarithm of the minimum angle of resolution.

*χ^2^ test (compared between 3 groups)

^†^Kruskal-Wallis test.

### Surgical techniques and postoperative treatment

All DMEK and DSAEK procedures were performed by one surgeon (TH). In phakic patients, cataract surgery with intraocular lens implantation was performed 1 month prior to EK.

#### DMEK

All surgeries were performed under retrobulbar anesthesia and a Nadbath facial nerve block. The pupil of the host eye was treated with miotic agents. The Descemet’s membrane graft was prepared and stained with 0.06% trypan blue dye. The Descemet’s membrane was peeled gently from the stroma so that an 8-mm-diameter flap of Descemet’s membrane with corneal endothelial cells was obtained. Four small asymmetric semicircular marks indicating the graft orientation were made on the edge of the graft. After creating two paracenteses on the corneal limbus, a 2.8-mm-wide corneoscleral tunnel was created at 12 o’clock. Peripheral iridotomy was performed at 6 o’clock using a 25-gauge vitreous cutter. An 8-mm-diameter descemetorhexis was created under air using a reverse Sinskey Hook. The 7.5-mm or 7.75-mm diameter graft was inserted using an intraocular lens inserter (WJ-60M®; Santen, Osaka, Japan), and all incisions were sutured using 10–0 nylon (Mani, Tochigi, Japan).[19] Then, the graft was unfolded by indirect manipulation with air and fluid. The anterior chamber was completely filled with air for 15 min. After 15 min, air-fluid exchange was performed until the anterior chamber was 80% filled with air. At the end of the procedure, 0.4 mg of subconjunctival betamethasone (Rinderon; Shionogi, Osaka, Japan) and 1.5% levofloxacin eye drops (Cravit; Santen, Osaka, Japan) were administered. Postoperative medications included 1.5% levofloxacin (Cravit), 0.1% betamethasone sodium phosphate (Sanbetasone; Santen), and 2% rebamipide ophthalmic solution (Mucosta; Otsuka, Japan, Tokyo), starting at 5 times per day for 3 months and tapered thereafter.

#### DSAEK

After local (retrobulbar anesthesia and a Nadbath facial nerve block), a 5.0-mm temporal corneal or corneoscleral incision was made. A 25G anterior chamber maintenance cannula was inserted in one side port. The recipient’s Descemet’s membrane was removed same as DMEK procedure. In most cases, the graft size was 7.75 mm or 8.0 mm. Pre-cut donor grafts were trephinated and the endothelial surface of the lenticle was coated with a small amount of viscoelastic material (Viscoat; Alcon). The donor tissue was gently inserted into the anterior chamber using a Busin glide (Asico) and Shimazaki DSAEK forceps (Inami, Tokyo, Japan). The pull-through technique was used to insert the donor graft. Air was carefully injected into the anterior chamber to unfold the graft. Fifteen minutes after the air injection, most of the air was replaced with balanced salt solution. Intraoperative and postoperative medication was the same as for DMEK.

### Examinations

Patients who had undergone DMEK or DSAEK were analyzed retrospectively. In addition to a standard ophthalmic examination, the BSCVA (logarithm of the minimum angle of resolution [logMAR]) and graft adaptation were evaluated preoperatively and at 1, 3, and 6 months postoperatively. Graft adaptation was assessed by observation using slit-lamp microscopy and anterior segment optical coherence tomography (AS-OCT; Casia SS-1000; Tomey, Nagoya, Japan). The AS-OCT equipment used was swept-source OCT, which uses a fast-wavelength scanning laser source and a balanced photodetector for spectrally resolved interferometric detection. The rotating 128-image three-dimensional scan measurement mode was used. The AS-OCT equipment automatically captured images when correct alignment in the x, y, and z directions was achieved.[20] After image capture, the instrument analyzed the 128 A-scan images of the anterior eye and allowed export of corneal elevation topographic data from the anterior and posterior surfaces. AS-OCT provided high-resolution corneal images and axial maps of the anterior and posterior surfaces, a keratometric value (3 mm), average corneal power within the central 3-mm diameter area, and central corneal thickness.

### Corneal higher-order aberrations

The corneal HOAs were evaluated preoperatively and at 3 and 6 months postoperatively at diameters of 6.0 mm using AS-OCT. We included not only HOAs but also spherical aberrations (SAs) and coma aberrations (Comas). All total, anterior, and posterior factors were analyzed. All subjects were examined until at least two sets of excellent images were obtained. Sixteen rotating AS-OCT scans were used to reconstruct three-dimensional models of the entire corneal structure. The Casia SS-1000 system corrected distortions in the AS-OCT images based on the refractive index of the anterior surface. A corneal specialist (TH) carefully checked all AS-OCT images to ensure that the surface digitalization recognized by the automated inbuilt software was correct. Zernike coefficients were calculated using Zernike analysis as previously reported.[9] In brief, the anterior and posterior corneal surfaces were reconstructed as a three-dimensional model from the corneal height data. The anterior, posterior, and total corneal aberrations at diameters of 4.0 mm and 6.0 mm were calculated separately using the installed ray tracing software (version 5.1). The refractive indices of the cornea and aqueous humor were set to 1.376 and 1.336, respectively. The wavefront aberration was expanded with normalized Zernike polynomials up to the 8th order. HOA was defined as the root mean square (RMS) of the 3rd to 8th order Zernike coefficients as a previous report. [15]

$$HOA=\sqrt{\sum_{j=3}^{20}(Z_{j})2}$$

$$Z_{J}=Z_{n}^{m}$$

$$j=\frac{n(n+2)+m}{2}$$

$$n=roundup\left[\frac{-3+\sqrt{9+8j}}{2}\right]$$

m=2j−n(n+2)

SA was defined as the RMS of Z_4_^0^ (spherical aberration) and Z_6_^0^ (secondary spherical aberration). Coma was defined as the RMS of Z_3_^-1^ and Z_3_^1^. [15]

$$SA=\sqrt{Z_{4}^{0^{2}}+Z_{6}^{0^{2}}}$$

$$Coma=\sqrt{Z_{3}^{{-1}^{2}}+Z_{3}^{1^{2}}}$$

### Definition of posterior topography grades

**Fig 2:**
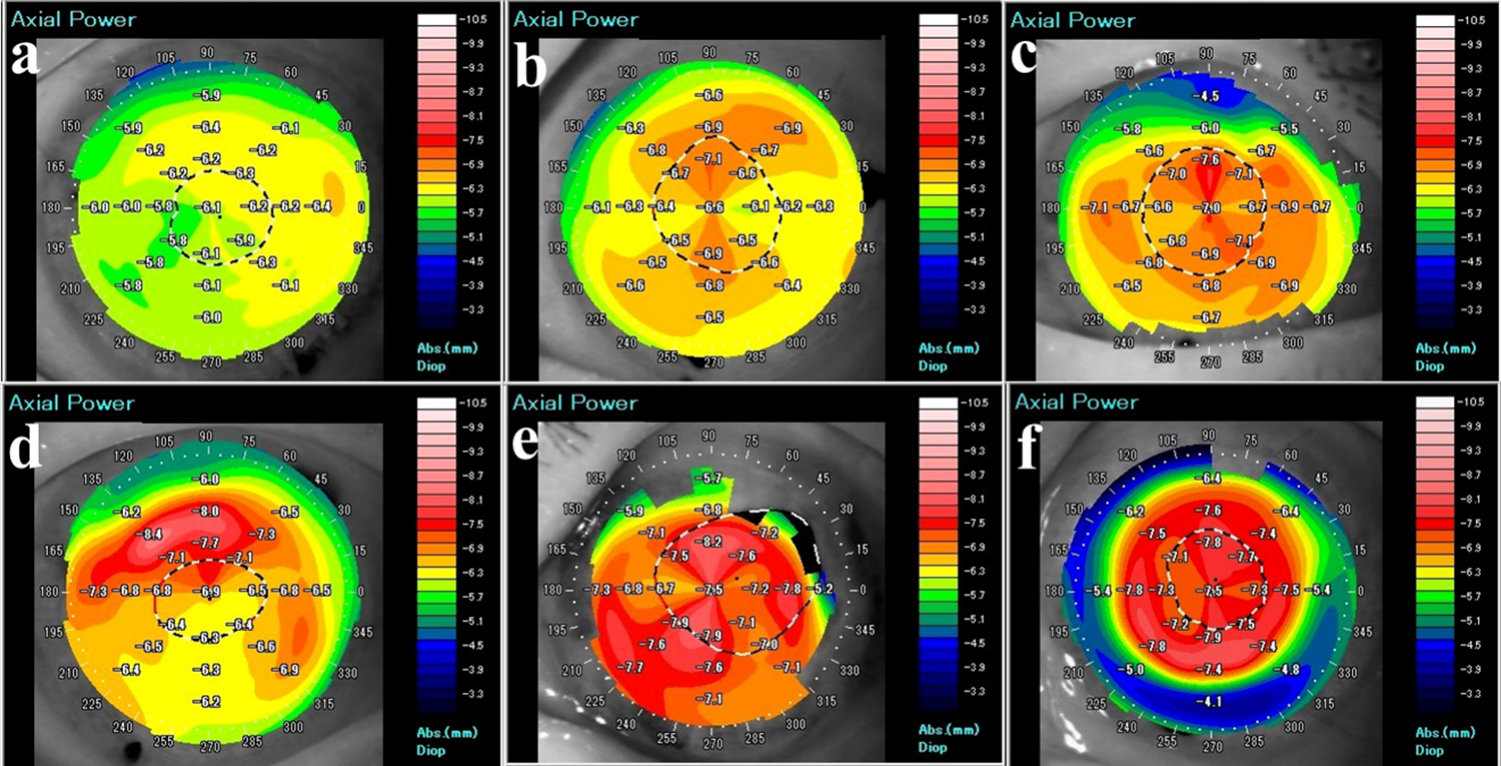
Posterior color grade after endothelial keratoplasty. Using anterior segment optical coherence tomography (AS-OCT), the posterior classification is used to score the cornea from 0 to 5 points according to PRP.
(A) Grade 0: Background consists of “cool” colors (green or blue).(B) Grade 1: Background is yellow.(C) Grade 2: Background is orange and total area of red is < 1/4(D) Grade 3: Background is orange and 1/4 < total area of red is < 1/2.(E) Grade 4: Background is orange and total area of red is > 1/2.(F) Grade 5: Background is red.

AS-OCT images of the posterior corneal surface were obtained from all patients after EK. Two blinded corneal specialists (TH, TY) used the AS-OCT images and posterior classification (Fig 2) to score the cornea from 0 to 5 points according to posterior refractive power (PRP); each cornea was scored 3 times in a masked fashion.

Scoring standards were as follows. In AS-OCT, PRP is shown in color. Each topographic color indicates refractive power; green or blue (PRP > -6.3 D), yellow (PRP = -6.3 D), orange (-6.9 D < PRP < -6.3 D), and red (PRP < -6.9 D). Cool colors indicate low PRP (flat) and hot/warm colors indicate high PRP (steep).

Grade 0: Background consists of “cool” colors (green or blue).
Grade 1: Background is yellow.
Grade 2: Background is orange and total area of red is < 1/4
Grade 3: Background is orange (-6.9 D < PRP < -6.3 D) and
1/4 < total area of red is < 1/2.
Grade 4: Background is orange and total area of red is > 1/2.
Grade 5: Background is red.

### Correlations between visual acuity, HOAs, and posterior color grades

The correlations between BSCVA and posterior color grade, type of surgery, HOAs, SAs, and Comas within a diameter of 6 mm were analyzed using Pearson’s correlation test. Type of surgery was classified as follows: DMEK (group 1) and DSAEK (group 2).

### Statistical analysis

The Wilcoxon test was used to compare mean values preoperatively and postoperatively where appropriate. The Mann–Whitney *U* test was used to compare mean values where appropriate between two groups. The male/female and right/left ratios were compared using the χ^2^ test. The Kruskal–Wallis test with Dunn’s multiple comparisons test was used to compare BSCVA, central corneal thickness, and HOAs between 3 groups. Pearson’s correlation analysis was used to evaluate the correlations between BSCVA and several other factors (HOAs, SAs, and Comas of the total cornea within a 6-mm diameter, posterior color grade, or type of surgery). All analyses were performed using StatView software (Abacus Concepts, Berkeley, CA, USA). A *P*-value < 0.05 was considered statistically significant.

## Results

### BSCVA

**Fig 3:**
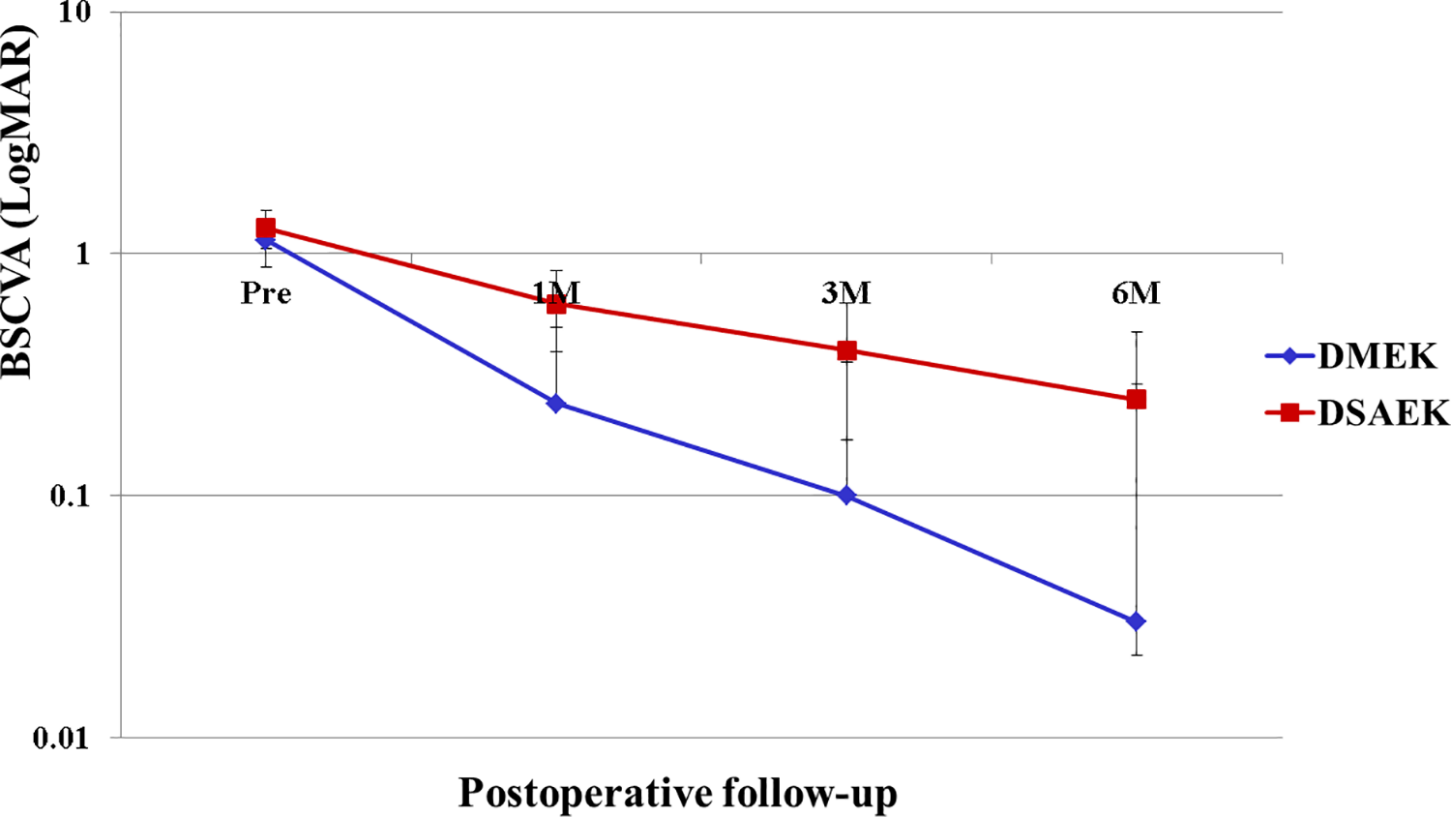
Comparison of BSCVA between DMEK and DSAEK. A statistically significant improvement in BSCVA is obtained in each group (*P* < 0.001, Wilcoxon rank sum test). There is also a statistically significant difference in BSCVA between the two groups at all postoperative examinations (*P = 0.020, **P < 0.001, and **P < 0.001 at 1, 3, and 6 months, respectively; Mann–Whitney *U* test). Abbreviations: BSCVA, best spectacle-corrected visual acuity; DMEK, Descemet’s endothelial membrane keratoplasty; DSAEK, Descemet’s stripping automated endothelial keratoplasty.

In the DMEK group, BSCVA improved from 1.01 ± 0.55 preoperatively to 0.23 ± 0.22 at 1 month, 0.09 ± 0.1 at 3 months, and 0.03 ± 0.06 at 6 months postoperatively. In the DSAEK group, BSCVA improved from 1.28 ± 0.43 preoperatively to 0.62 ± 0.26 at 1 month, 0.40 ± 0.19 at 3 months, and 0.25 ± 0.16 at 6 months. There was a statistically significant improvement in BSCVA in each group (*P* < 0.001; Wilcoxon rank sum test). Notably, the difference in BSCVA between the 2 groups was statistically significant at all postoperative follow-up examinations (*P* = 0.02 at 1 month, *P* < 0.001 at 3 months, and *P* < 0.001 at 6 months; Mann–Whitney *U* test; Fig 3).

### Corneal HOAs

Table 2 shows the mean HOAs of the Zernike terms for the 6-mm diameters in each group. Compared with the healthy controls, all HOA parameters, including HOAs, SA, and Comas (total/anterior/posterior, 6.0 mm) were significantly higher in the DMEK and DSAEK groups preoperatively (*P* < 0.05; Dunn’s multiple comparison test). There were no significant differences in HOAs (total/anterior/posterior, 6.0 mm) between the DMEK and DSAEK groups preoperatively. However, posterior HOAs at 6 mm in the DMEK group were significantly lower than those in the DSAEK group at any time postoperatively (*P* < 0.05; Dunn’s multiple comparison test). Postoperative total/anterior/posterior SAs at 6.0 mm were significantly lower in the DMEK group than those in the DSAEK group at all postoperative assessments (*P* < 0.05; Dunn’s multiple comparison test). Posterior Comas at 6.0 mm were significant lower in the DMEK group than those in the DSAEK group at 6 months (*P* = 0.036; Dunn’s multiple comparison test).

**Table 2:** Corneal aberrations in the central 6.0-mm zones in descemet membrane endothelial keratoplasty, descemet’s stripping automated endothelial keratoplasty, and control groups.

		Pre		3M		6M	
	Control	DMEK	DSAEK	DMEK	DSAEK	DMEK	DSAEK
HOA (6mm)							
Total	0.19±0.05	0.76±0.47	1.03±0.47	0.46±0.17	0.83±0.39	0.45±0.19	0.69±0.28
		p^DMEK/CT^	**<0.001**	p^DMEK/CT^	**0.001**	p^DMEK/CT^	**0.001**
		p^DSAEK/CT^	**<0.001**	p^DSAEK/CT^	**<0.001**	p^DSAEK/CT^	**<0.001**
		p^DMEK/DSAEK^	1.000	p^DMEK/DSAEK^	0.082	p^DMEK/DSAEK^	0.315
Anterior	0.19±0.07	0.76±0.46	1.05±0.50	0.43±0.15	0.79±0.37	0.43±0.17	0.70±0.28
		p^DMEK/CT^	**<0.001**	p^DMEK/CT^	**<0.001**	p^DMEK/CT^	**0.004**
		p^DSAEK/CT^	**<0.001**	p^DSAEK/CT^	**<0.001**	p^DSAEK/CT^	**<0.001**
		p^DMEK/DSAEK^	1.000	p^DMEK/DSAEK^	0.067	p^DMEK/DSAEK^	0.189
Posterior	0.07±0.05	0.22±0.14	0.22±0.09	0.15±0.06	0.27±0.12	0.12±0.04	0.23±0.09
		p^DMEK/CT^	**<0.001**	p^DMEK/CT^	**<0.001**	p^DMEK/CT^	**0.002**
		p^DSAEK/CT^	**<0.001**	p^DSAEK/CT^	**<0.001**	p^DSAEK/CT^	**<0.001**
		p^DMEK/DSAEK^	1.000	p^DMEK/DSAEK^	**0.024**	p^DMEK/DSAEK^	**0.034**
SA (6mm)							
Total	0.13±0.04	0.44±0.26	0.58±0.28	0.23±0.10	0.42±0.21	0.19±0.08	0.34±0.13
		p^DMEK/CT^	**<0.001**	p^DMEK/CT^	**0.047**	p^DMEK/CT^	0.214
		p^DSAEK/CT^	**<0.001**	p^DSAEK/CT^	**<0.001**	p^DSAEK/CT^	**<0.001**
		p^DMEK/DSAEK^	0.421	p^DMEK/DSAEK^	**0.047**	p^DMEK/DSAEK^	**<0.001**
Anterior	0.14±0.05	0.42±0.24	0.58±0.30	0.23±0.10	0.42±0.21	0.20±0.11	0.35±0.13
		p^DMEK/CT^	**<0.001**	p^DMEK/CT^	0.119	p^DMEK/CT^	0.303
		p^DSAEK/CT^	**<0.001**	p^DSAEK/CT^	**<0.001**	p^DSAEK/CT^	**<0.001**
		p^DMEK/DSAEK^	0.408	p^DMEK/DSAEK^	**<0.001**	p^DMEK/DSAEK^	**<0.001**
Posterior	0.04±0.01	0.14±0.10	0.12±0.05	0.09±0.03	0.17±0.06	0.08±0.02	0.15±0.05
		p^DMEK/CT^	**<0.001**	p^DMEK/CT^	**0.002**	p^DMEK/CT^	**0.005**
		p^DSAEK/CT^	**<0.001**	p^DSAEK/CT^	**<0.001**	p^DSAEK/CT^	**<0.001**
		p^DMEK/DSAEK^	0.423	p^DMEK/DSAEK^	**0.021**	p^DMEK/DSAEK^	**0.013**
Coma (6mm)							
Total	0.15±0.06	0.62±0.40	0.85±0.40	0.39±0.16	0.67±0.36	0.39±0.19	0.59±0.28
		p^DMEK/CT^	**<0.001**	p^DMEK/CT^	**<0.001**	p^DMEK/CT^	**<0.001**
		p^DSAEK/CT^	**<0.001**	p^DSAEK/CT^	**<0.001**	p^DSAEK/CT^	**<0.001**
		p^DMEK/DSAEK^	0.642	p^DMEK/DSAEK^	1	p^DMEK/DSAEK^	0.994
Anterior	0.16±0.06	0.62±0.42	0.86±0.42	0.36±0.14	0.64±0.37	0.37±0.16	0.59±0.28
		p^DMEK/CT^	**<0.001**	p^DMEK/CT^	**<0.001**	p^DMEK/CT^	**<0.001**
		p^DSAEK/CT^	**0.003**	p^DSAEK/CT^	**<0.001**	p^DSAEK/CT^	**<0.001**
		p^DMEK/DSAEK^	0.558	p^DMEK/DSAEK^	1	p^DMEK/DSAEK^	1
Posterior	0.03±0.01	0.17±0.10	0.18±0.08	0.12±0.07	0.20±0.11	0.08±0.04	0.17±0.08
		p^DMEK/CT^	**<0.001**	p^DMEK/CT^	**<0.001**	p^DMEK/CT^	**0.002**
		p^DSAEK/CT^	**<0.001**	p^DSAEK/CT^	**<0.001**	p^DSAEK/CT^	**<0.001**
		p^DMEK/DSAEK^	1	p^DMEK/DSAEK^	0.119	p^DMEK/DSAEK^	**0.036**

DMEK, Descemet’s membrane endothelial keratoplasty; HOA, higher-order aberrations; SA, spherical aberrations; Coma, coma aberrations

### Posterior color grade using AS-OCT

The topography maps of the posterior cornea after EK were classified using AS-OCT. Typically, the posterior of an eye that has undergone DSAEK is red. The posterior color grade was 2.3 ± 1.2, 1.4 ± 0.9, and 1.2 ± 1.0 at 1, 3, and 6 months, respectively, in the DMEK group and 4.4 ± 0.8, 3.6 ± 1.0, and 2.8 ± 1.1, respectively, in the DSAEK group. The posterior color grades in the DMEK group were significantly lower than those in the DSAEK group at all postoperative examinations (*P* < 0.001, at 1, 3, and 6 months, respectively; Mann–Whitney *U* test). In normal corneas, the grade was 0.44 ± 0.50.

### Correlations between visual acuity, HOAs, and posterior color grades

There was a positive correlation between BSCVA and posterior color grade (Table 3). The BSCVA had a significant positive correlation with HOAs and SAs at the 6.0-mm zones of the total cornea and the anterior and posterior surfaces. BSCVA had a significant positive correlation with Comas at the 6.0-mm zones of the total cornea and the posterior surfaces. The BSCVA had no correlation with Comas at the 6.0-mm zones of the anterior cornea. Posterior color grades and type of surgery (DMEK or DSAEK) were other important factors that were correlated with BSCVA.

**Table 3:** Correlations between visual acuity, HOAs, and posterior color grades.

	R	p
Color grade	0.664	**<0.001**
Total HOA (6mm)	0.555	**<0.001**
Anterior HOA (6mm)	0.300	**0.004**
Posterior HOA (6mm)	0.493	**<0.001**
TotalSA (6mm)	0.619	**<0.001**
Anterior SA (6mm)	0.405	**<0.001**
Posterior SA (6mm)	0.462	**<0.001**
Total Coma (6mm)	0265	**0.012**
Anterior Coma (6mm)	0.036	0.735 (NS)
Posterior Coma (6mm)	0.401	**<0.001**
DMEK (1) or DSAEK (2)	0.353	**<0.001**

## Discussion

In this study, we demonstrated that HOAs are increased in comparison with a control group after EK, and more so after DSAEK, than after DMEK. The DMEK group had significantly lower HOAs than the DSAEK group. Further, HOAs were inversely correlated with BSCVA after EK, suggesting that both posterior color grades and corneal HOAs could be clinical indices to explain poor visual acuity after EK.

These findings are in line with previous reports on the impact of HOAs on visual acuity after DMEK.[4, 5] Rudolph et al reported that DMEK was superior to DSAEK with respect to less posterior corneal surface HOAs and better BSCVA,[4] while van Dijk et al found that optical quality was improved after DMEK in comparison with controls.[5] In the study by van Dijk et al, the anterior HOA, posterior HOA, and backscattered light values were higher in eyes after DMEK than in control eyes. These authors also demonstrated that preoperative BSCVA, patient age, anterior backscattered light, and anterior HOAs had a negative impact on postoperative BSCVA.[5] Because AS-OCT does not evaluate anterior backscattered light, we could not obtain this information. In the present study, BSCVA was not correlated with preoperative BSCVA or patient age. To the best of our knowledge, there have been no clinical reports on the apparent correlation between visual acuity and HOAs of the posterior surface after DMEK.

In the present study, BSCVA was correlated not only with HOAs of the posterior surface but also with those of the total cornea and anterior surface. Moreover, the topographic color grading system makes it easy to determine and diagnose the severity of the irregularity on the posterior corneal surface in clinical practice. Therefore, it is likely that the posterior color grades used in the current study could predict visual acuity in patients after EK and diagnose the reason for poor visual acuity.

In general, HOAs of the anterior surface are compensated for by the posterior cornea in eyes with a healthy cornea and in eyes with corneal disorders, which in turn reduces total corneal HOAs by 4% to 33%.[21, 22] In our study, the total corneal HOAs were similar to the HOAs of the anterior surface in normal control eyes and in eyes that underwent DSAEK. Therefore, the negative influence of the posterior surface on HOAs of the total cornea may be negligible. However, among the corneal HOAs after EK, the SA value at 6.0 mm was relatively large when compared with total HOAs, especially in eyes after DSAEK. In our study, the amount of SA at 6.0 mm was 0.13 μm in normal eyes, 0.19 μm at 6 months after DMEK, and 0.34 μm at 6 months after DSAEK. It is reasonable that the SA value after DMEK would be much smaller than that after DSAEK, because DMEK does not include graft stroma and a meniscus-shaped stroma may induce SA in eyes that have undergone DSAEK. It has been well documented that less SA is associated with improved contrast sensitivity.[7] SA is an aberration that represents the difference in refractive power between the paraxial and peripheral areas, which can be corrected by aspheric intraocular lenses.[23] Therefore, reduction of SA using an aspheric intraocular lens could potentially improve visual acuity after EK. This possibility should be investigated further in a prospective study.

**Fig 4:**
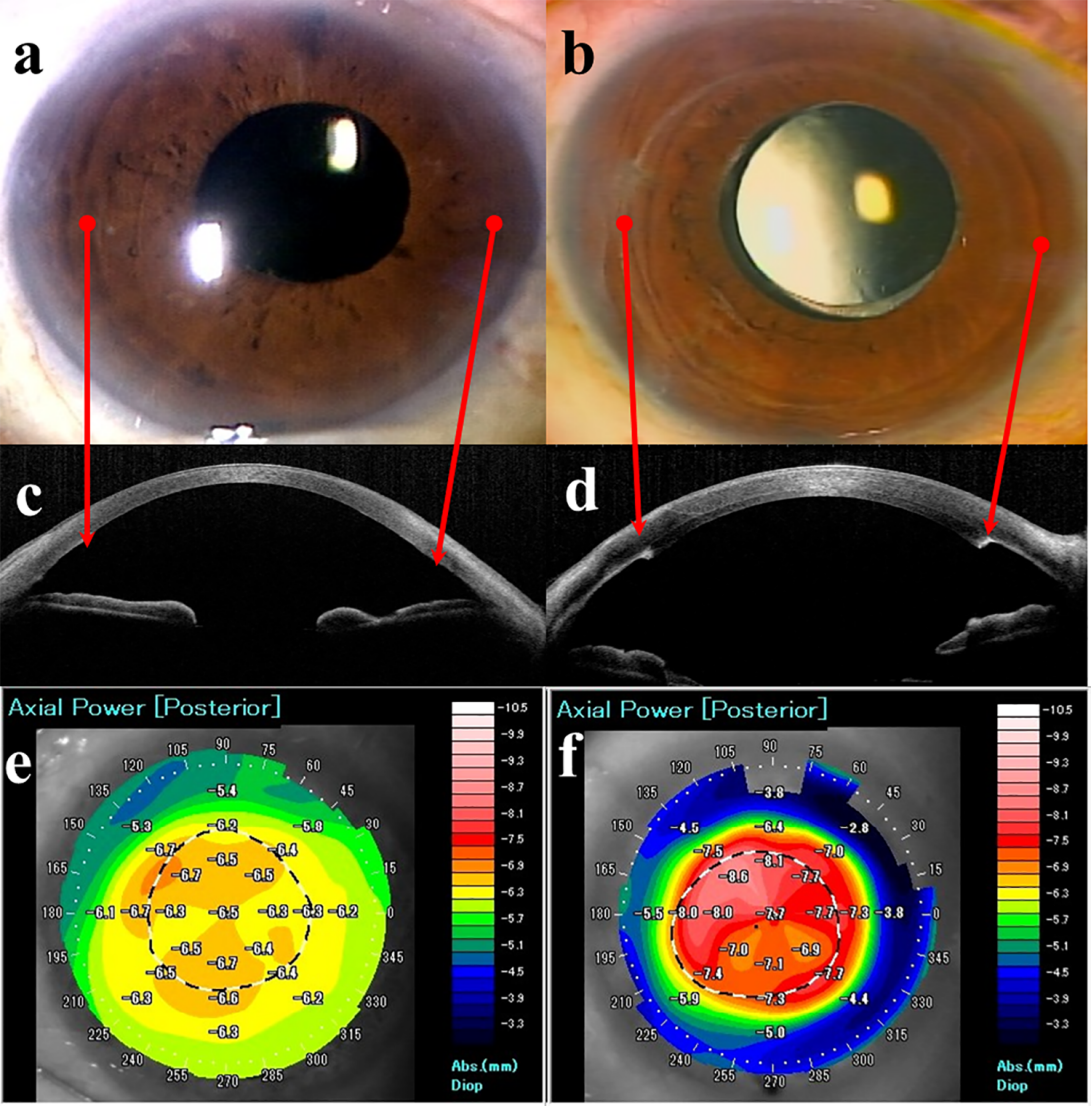
Representative cases of slit-lamp microscopy and AS-OCT after endothelial keratoplasty. (A) Slit-lamp photograph 6 months after DMEK. (B) Slit-lamp photograph 6 months after DSAEK. (C) AS-OCT section 6 months after DMEK. (D) AS-OCT section 6 months after DSAEK. (E) Posterior map using AS-OCT 6 months after DMEK. (F) Posterior map using AS-OCT 6 months after DSAEK. Postoperatively, a DMEK eye is difficult to distinguish from a normal eye after cataract surgery (A). However, we can see the scarring edge of the DSAEK graft (B). An AS-OCT section shows the natural posterior curvature in the DMEK eye (C), whereas the DSAEK eye has a meniscus-shaped posterior protrusion in the central cornea (D). The posterior color is cool in the DMEK eye (E), but red in the central cornea of the DSAEK eye (F). The arrows show the virtual peripheral edges of the DMEK and DSAEK grafts. Abbreviations: AS-OCT, anterior segment optical coherence tomography; DMEK, Descemet’s endothelial membrane keratoplasty; DSAEK, Descemet’s stripping automated endothelial keratoplasty.

Given that DMEK only involves transplantation of Descemet’s membrane and endothelial cells, we expected that eyes would retain almost normal physiologic corneal curvature and SA value postoperatively. However, the SA value after DMEK was almost twice that in the normal control group. To assess the corneal shape in detail, we analyzed the posterior surface topography maps and classified them using posterior color grades. Our analyses revealed large interindividual differences in the posterior color map after EK, and even after DMEK. This finding is clinically relevant because there is a strong correlation between posterior color grade and BSCVA after EK. Although eyes that had undergone DMEK had a lower posterior color grade than those that had undergone DSAEK (Fig 4), some patients with poor visual acuity had a higher color grade (grade 2), as shown in Fig 1. We postulate that the posterior color grade may have been associated with the extent of dehydration of the corneal stroma in eyes that had undergone DMEK because the central corneal thickness was around 450 μm. After DMEK, acute improvement of corneal edema only occurs in the central area and the posterior surface becomes steep. Theoretically, this can lead to a hyperopic shift and a larger SA value after DMEK.

Although DMEK is supposed to be anatomically perfect keratoplasty, physiologic surface parallelism is abnormal postoperatively. This postoperative optical abnormality might be a limitation in some eyes after DMEK. Inoue et al reported a swirling pattern of horizontal water migration in the cornea in human and rabbit eyes,[24, 25] which is identical to the anatomic pattern of the corneal nerve. Because DMEK replaces dysfunctional endothelium, key factors in need of investigation include an uneven distribution of endothelial cell density. Although recent clinical studies have reported good visual outcomes after hemi-DMEK,[26, 27] the visual acuity after hemi-DMEK seems to be relatively worse than that after conventional DMEK.[28] Future comprehensive studies on the corneal shape, endothelial cell distribution, the corneal nerve, and extent of edema may identify the reason for the posterior color map patterns. Further, less invasive treatment, such as corneal endothelial cell injection therapy, might resolve this problem.[29]

Our study has some limitations, including a small sample size, a retrospective design, and lack of evaluation of forward scattering. Because of our small sample size, we used a combined analysis of the correlation between visual outcome and some parameters in each group, and we found a strong correlation between BSCVA and posterior color grade. AS-OCT cannot evaluate forward scattering, so further prospective studies of HOAs and forward scattering after EK in are needed in larger numbers in Asian patients. Although the etiology of bullous keratopathy differs between Asian and Western countries, quality of vision is better after DMEK than after DSAEK. The visual outcomes are not inferior to those in many reports from Western countries, where FECD is a major cause of DMEK. In our study, about one third of our patients in each group had ALI-induced BK, which is a common indication for EK in Japan.[30]

In conclusion, there are fewer topographic changes after DMEK than after DSAEK. However, the visual outcome strongly depends on the topographic changes at the posterior cornea or anterior and posterior HOAs. Although during the early postoperative period, topographic color grade was high, the grade could gradually resolve with time. Therefore, postoperative assessment of topographic changes at the posterior cornea would be useful for evaluating the visual function after EK.

## Supporting information

S1 Table Patient data for all tables (Tables 1, 2 and 3), dunn’s test for Table 2, and correlations among visual acuity, high-order abberations (HOAs), and posterior color grade in control group, DMEK group, and DSAEK group. (XLSX)
